# Collaborative Social-Epidemiology: A Co-analysis of the Cultural and Structural Determinants of Health for Aboriginal Youth in Victorian Schools

**DOI:** 10.3390/ijerph18168674

**Published:** 2021-08-17

**Authors:** Joanne Nicole Luke, Alister Thorpe, Carlina Black, Lisa Thorpe, David Thomas, Sandra Eades, Kevin Rowley

**Affiliations:** 1 School of Population and Global Health, The University of Melbourne, Carlton 3053, Australia; ahthorpe@unimelb.edu.au (A.T.); sandra.eades@curtin.edu.au (S.E.); j.luke2@student.unimelb.edu.au (K.R.); 2 The Victorian Aboriginal Child Care Agency, Preston 3072, Australia; C.Black@latrobe.edu.au; 3 Bubup Wilam-Aboriginal Child and Family Centre, Thomastown 3074, Australia; lisa.thorpe@bubupwilam.org.au; 4 Menzies School of Health Research, Charles Darwin University, Darwin 0810, Australia; David.Thomas@menzies.edu.au; 5 Curtin Medical School, Curtin University, Bentley 6102, Australia

**Keywords:** Aboriginal, health and wellbeing, cultural determinants, structural determinants, adolescent, Victoria

## Abstract

Social-epidemiology that excludes Aboriginal voices often fails to capture the full and complex social worlds of Aboriginal people. Using data from an existing co-designed Victorian government Adolescent Health and Wellbeing Survey (2008/9), we worked with Aboriginal organizations to identify data priorities, select measures, interpret data, and contextualize findings. Using this participatory co-analysis approach, we selected “cultural” and “structural” determinants identified by Aboriginal organizations as important and modelled these using principal component analysis. Resulting components were then modelled using logistic regression to investigate associations with “likely being well” (Kessler-10 score < 20) for 88 Aboriginal adolescents aged 11–17 years. Principal component analysis grouped 11 structural variables into four components and 11 cultural variables into three components. Of these, “grew up in Aboriginal family/community and connected” associated with significantly higher odds of “likely being well” (OR = 2.26 (1.01–5.06), *p* = 0.046). Conversely, “institutionally imposed family displacement” had significantly lower odds (OR = 0.49 (0.24–0.97), *p* = 0.040) and “negative police contact and poverty” non-significantly lower odds (OR = 0.53 (0.26–1.06), *p* = 0.073) for “likely being well”. Using a co-analysis participatory approach, the voices of Aboriginal researchers and Aboriginal organizations were able to construct a social world that aligned with their ways of knowing, doing, and being. Findings highlighted institutionally imposed family displacement, policing, and poverty as social sites for health intervention and emphasized the importance of strong Aboriginal families for adolescents.

**Please note:** Throughout this paper, the term Aboriginal is used describe the first people living in Victoria, who come from 250+ First Nations of territories now claimed as Australia. We use this as a collective term and recognize that Aboriginal is a term imposed by Europeans and not without contention. However, Aboriginal is a term widely used by Aboriginal organizations themselves in Victoria. Throughout this paper, individual First Nations are also referred to where individuals use these terms to self-describe identities of nationhood. The term “Stolen Generations” refers to Aboriginal children and their descendants who were forcibly removed from their families prior to the 1970s as part of official Australian government policies.

## 1. Introduction

Epidemiology, translating to “the study of what is upon the people”, is often criticized for its failure to capture the often complicated relationships between multiple and interacting determinants impacting “upon” people’s lives [1]. Although epidemiology relating to Aboriginal populations has in recent years increasingly taken a social approach to measuring health and its broader social determinants, including increasing focus on strengths-based approaches, much social-epidemiology continues to be limited by a failure to centre Aboriginal people, organizations, and knowledges in “the study of what is upon Aboriginal people”. This absence of Aboriginal voices has meant that many important social and cultural dimensions that make up the realities of Aboriginal people’s lives and prioritized as important by Aboriginal people are not attended to in epidemiology.

### 1.1. Epidemiology and the Individual Focus

Epidemiology has been defined as “the study of the distribution and determinants of health-related states or events in specified populations, and the application of this study to control of health problems” [2] (p. 3).

We know that the origins of epidemiology are rooted in the ecological, in the understanding that social factors determine the health of populations [3]. The formative public health efforts of John Snow, William Farr, Edwin Chadwick, and Friedrich Engels in the mid-1800s all demonstrated disease as a consequence of social class injustices, as determined by factors external to the individual [4,5]. But with the development of infectious disease epidemiology in the 1800s and chronic disease modelling in the mid-1900s, explanatory modelling shifted away from a social to a biological frame [6]. In considering epidemiology relating to Aboriginal populations, this evolved in the twentieth century, when there was a vigour for collecting anthropological data about individual bodies and minds [7]. Epidemiology relating to Aboriginal people started at this point of knowing the individual.

Scholarship by Brough (2013), and Walter (2010) has challenged the utility of descriptive bioepidemiology that maintains a narrow focus on the individual or the Aboriginal community [1,8]. These critics highlight that framing health as a consequence of self- perpetuating deficits of individual- or group-level biology and behaviour, without consideration to the environments with which biology interacts and where communities live, is problematic, particularly when those individual Aboriginal bodies, minds, and cultures are exposed to social, political, and economic structuring that comes from colonization and being raced [9]. This narrative of problematic individuals is most pronounced for Aboriginal adolescent health. Epidemiology involving Aboriginal adolescents to date has largely adopted a victim-blaming narrative that sees young Aboriginal people engaged in “risky behaviours” and making “poor life choices”, particularly when it comes to sexual health. A systematic review by Azzopardi et al. 2013 that examined health research nationally identified that research relating to Aboriginal adolescent health largely maintained a focus on the 11 risks of tobacco, alcohol, illicit drugs, high body mass, physical inactivity, low fruit and vegetable intake, high blood pressure, high cholesterol, unsafe sex, child sexual abuse, and intimate partner violence, with limited engagement into the contexts and mechanisms that give rise to these risks. Social-epidemiology allows us to engage with some of these important contexts and mechanisms.

This absence of a comprehensive picture of Aboriginal adolescent health is a real barrier to enabling effective policy [10]. Data on the health of Aboriginal adolescents and its social determinants are instrumental to health planning, policy, resourcing, and evaluation.

### 1.2. Social Epidemiology

Recent decades have seen a re-emergence of the “social determinants approach”, and for epidemiology, this has seen the development of ecosocial (systems) theory, where conceptualizations of health are turned to “who and what is responsible for population patterns of health, disease, and wellbeing, as manifested in present, past, and changing social inequalities in health” [11] (p. 694). Aboriginal populations have strong understandings of who and what is responsible for health [12]. Aboriginal people and communities have long asserted that health is socioculturally determined by Australian-society-level structural determinants and by community-level cultural determinants, and that the application of this knowledge to social-epidemiology should be straightforward [12,13,14]. However, most research has taken a dominant understanding to social determinants that has assumed a universality of Australian dominant social contexts, with limited attention to measures or indicators that capture the unique social experiences of Aboriginal populations.

### 1.3. Aboriginal People in Victoria—Local Ways of Knowing the Social Determinants

There is over 50 years of research and policy by Aboriginal communities in Victoria that speaks to Aboriginal understandings of health and its unique social determinants [13,15,16]. This includes numerous research projects that have challenged the transposition of dominant social indicators onto Aboriginal populations, highlighting that Aboriginal people participate in their own societies (i.e., communities) and participate differently in dominant societies (i.e., are marginalized). For example, Tynan et al. (2005) and colleagues from Aboriginal communities across the Goulburn–Murray Rivers region highlighted how the social lives of Aboriginal people are different: “Not only are there distinct cultural values contributing to different social processes in Koori communities, but the ongoing social marginalization of Koori people from mainstream society contributes to a substantially different social domain” [16] (p. 2). Similarly, Aunty Joan Vickery, a Gunditjmara Elder and long-time health advocate, highlighted that dominant social determinants are unlikely to capture the full and complex social realities of Aboriginal lives, especially when they include those that are imposed and of a colonizing or assimilative nature [15].

There are existing conceptual research frameworks that identify and describe social determinants from the “insider” perspective of Aboriginal people living in Victoria [15,17,18,19,20,21,22]. These have largely taken an ecological perspective that links family, community, and wider social systems to Aboriginal health. The most widely recognized of these, by Aboriginal academic Dr. Graham Gee et al. (2010), largely frames Aboriginal cultural determinants as health assets and the structural determinants of Australian society as contributing to poorer health.

By structural determinants, these models refer to the sociocultural, historical, and political contexts as well as the structural mechanisms that give rise to health inequities [23]. For Aboriginal people, dominant institutions, systems of governance, state and federal policies, welfare states, and dominant value systems are examples of sociocultural, historical, and political contexts, while ongoing racism and colonization form fundamental structural mechanisms that stratify and determine group access to resources and positions of power. By cultural determinants, these models refer to group-level determinants specific to Aboriginal peoples, often defined by them as the “aspects of culture which foster resilience that are protective of health, and that contribute to our identity and unique place within the Australian polity” [24] (p. 3).

Although structural and cultural determinants feature prominently in Aboriginal health policy and research frameworks, they have long been poorly conceptualized and measured in epidemiological analysis. Recently, there have been increasing calls to strengthen the evidence that cultural determinants are connected to health outcomes [24] as well as calls for research to critically engage with the broader social and political dimensions of health [25]. Although we recognize increasing work in this area to at least measure cultural determinants, we remain acutely aware of the lack of appropriate data, measures, and methodologies for quantifying the social dimensions of Aboriginal lives [26,27].

Here we drew on one of the very few government surveys in Victoria that was co-designed by local Aboriginal organizations and included community-identified social measures of structural and cultural determinants specific to Aboriginal people–the Victorian Adolescent Health and Wellbeing survey, also known as the HOWRU Survey.

### 1.4. The Victorian Adolescent Health and Wellbeing (HOWRU) Survey

The HOWRU survey was designed in 2008 by the Centre for Adolescent Health at the Royal Children’s Hospital in Melbourne on behalf of the Victorian government Department of Education and Early Childhood Development (DEECD). This survey included an Aboriginal module that was designed by the Onemda Koori Health Unit (Onemda) at the University of Melbourne and the Institute of Koorie Education at Deakin University [28]. It was developed under the governance of an Aboriginal steering committee, comprising Aboriginal organizations including the Victorian Aboriginal Child Care Agency (VACCA) and the Victorian Aboriginal Community Controlled Health Organisation (VACCHO), and two staff at the Royal Children’s Hospital. The resulting survey instrument had 32 items relating to the areas of Aboriginal identity, aspirations, success, family and community, service use, connection, participation in cultural activities, contact with dominant institutions, school environments, and discrimination and racism [28]. In addition to the Aboriginal module, the wider HOWRU survey included items relevant to all Victorian populations relating to demographics, school experiences, family structure and relationships, health and wellbeing, personal experiences, neighbourhood amenities, access to services, and safety. The Aboriginal module did not include a specific construct measure for Aboriginal health and wellbeing, but the wider HOWRU survey included standardized measures including the Kessler-10 psychological distress score.

In total, 10,424 adolescents from years 7, 9, and 11 across Victorian government, Catholic, and independent schools in metropolitan Melbourne and regional Victoria consented and participated in the 2008–2009 survey. Detailed methods for the wider HOWRU survey have previously been reported [29] and data have been extensively published for the broader cohort [30,31,32,33,34,35]. However, to date, only basic descriptive data have been reported for Aboriginal participants within a government document, “The state of Victoria’s children 2009: Aboriginal children and young people in Victoria”.

In this paper, we describe a co-analysis of the HOWRU survey. In doing so, we reframe the traditional power relations in epidemiology through a participatory co-analysis wherein Aboriginal organizations were active in identifying the research question, designing the analysis, identifying the variables, and framing and reporting the findings. This co-design process was consistent with an Indigenous rights based and data sovereignty agenda, and in line with key recommendation that the HOWRU survey be analysed by Aboriginal people and their representative bodies [28,36].

## 2. Materials and Methods

### 2.1. Ethical Considerations

The University of Melbourne Ethics Committee approved the project (ID:1443502.1). Permissions to access and analyse data were provided by the Department of Education and Early Childhood Development. In line with local protocols, an application for research partnership was submitted to VACCHO and approved.

At project commencement, Terms of Reference were devised and agreed to by all coauthors to guide the partnership and co-analysis. These terms outlined expectations relating to ethical conduct, community protocols, roles and responsibilities, reporting and knowledge translation, authorship, intellectual property, and student involvement.

### 2.2. Engagement, Collaboration, and Co-analysis Process

Data analysis was led by an Aboriginal (Alywarre/Stolen Generation) Ph.D. Student (J.L.), who alongside Gunai, Yorta Yorta, and Gunditjmara researchers (A.T.) engaged staff at three Aboriginal Community Controlled Health Organizations (Victorian Aboriginal Child Care Agency (VACCA), Victorian Aboriginal Community Controlled Health Organisation (VACCHO) and Bubup Wilam Early Learning Centre to co-analyse precollected cross-sectional survey data relating to Aboriginal adolescent health in Victoria. Representatives from Aboriginal organizations were engaged to allow for local understandings of sociocultural realities and lived experiences in the Victorian community to guide this research.

Co-analysis was conducted between 2015–2021 and involved three broad meetings with the Aboriginal researchers and staff from Aboriginal organizations. In these meetings representatives from Aboriginal organizations–1. identified organizational priorities and data needs; 2. designed and framed the analysis by selecting variables and outcomes that aligned best with local sociocultural understandings of health and wellbeing; and 3. interpreted data, contextualized findings and decided on processes for dissemination of findings.

Both the priorities of Aboriginal researchers and staff from Aboriginal-community-controlled organizations are reflected in this work.

### 2.3. Study Populaiton

In total 88 (0.8% of adolescents sampled) self-identified as Aboriginal and completed the Aboriginal module and have been included in our co-analysis. In recognizing the small sample size, our analysis is compromised by limited statistical power. However, we emphasize here the collaborative process for data-analysis rather than findings alone.

### 2.4. Demographic Variables

Age was measured as age at last birthday (in years). Gender was collected as a self-reported binary (male/female). A binary location variable was created using postcode coded to the Australian Bureau of Statistics 2011 Australian Statistical Geography Standard (ASGS) in regard to remoteness. Because of small sample size, inner regional and outer region classifications were merged, and a dichotomous variable, “major city” or “regional”, was created.

### 2.5. Explanatory Variables

The collaborative team identified which cultural and structural determinants fit with the work they did and with their understanding of social determinants. Because of the novel nature of these measures, we include how these were collected and measured as appendices. Measures were dichotomized to present the descriptive characteristics of the population (see Appendix A Table A1 and Table A2), but for principal component analysis, categorical variables with all responses were used where possible.

### 2.6. Outcome Measure

An inverted version of the Kessler-10 psychological distress questionnaire (K-10) was used [37]. Kessler-10 scores were dichotomized into “likely to be well”, corresponding to a score of 10–19, and “likely to have psychological distress”, corresponding to a score of 20–50. “Likely being well” was the outcome of interest and was selected to focus on youth doing well, a salutogenic approach to disrupt narratives of problemed adolescents. This approach has been used for Aboriginal populations elsewhere to describe subsets of Aboriginal populations doing well [38,39,40].

### 2.7. Statistical Analysis

Age has been summarized in terms of means and standard deviation. All categorical data were expressed as frequencies (percentages) and 95% CI. For categorical data, the χ^2^ test was used to assess difference between the groups “likely being well” and “likely to have psychological distress”. Rate ratios (RR) were calculated as “likely being well”/”likely to have psychological distress”, and confidence intervals for RR were calculated using MedCalc (MedCalc software, Ostend, Belgium). All other analyses were completed using SPSS Statistics version 27 (SPSS Inc., Chicago, IL, USA).

PCA was used to group highly correlated variables into a smaller number of “components”. Correlations between variables were determined using a correlation matrix. PCA was used to expose latent clustering of variables in the population. We ran two PCA models, one for cultural determinants and one for structural determinants, because of a small sample size (*n* = 88), as model stability would be compromised by the inclusion of too many variables [41,42].

Because of constraints on number of variables able to be included, we used a correlation matrix to assess the strengths of intercorrelations among the cultural and structural determinants. Where variables had poor correlation (no correlations > 0.3), they were excluded from PCA; these included neighbourhood attachment, high housing transition, access to basic services, health access, sees members of extended family, having Aboriginal friends, speaking language, participation in Aboriginal ceremonies, going to Aboriginal funerals or sorry business, and going to Aboriginal organizations, which were used only in univariate analysis. The suitability of data for PCA was assessed by testing appropriateness of sample size using Kaiser–Meyer–Olkin (KMO) value and the Bartlett test of sphericity [43].

The PCA used an Oblimin rotation given that correlation matrices revealed that variables were highly correlated. Components were extracted from PCA on the basis of an eigenvalue > 1. To interpret PCA models, loadings (correlations) greater than 0.4 were considered meaningful. In addition, components arising from PCA modelling were culturally validated through the co-analysis process where the collaborative members identified whether the components produced made sense and captured the social experiences of the communities they worked with. Components produced using PCA were used as continuous variables. Linear regression was used to assess associations between the cultural and structural PCA components, with scatter plots uses to assess suitability for linear regression.

Components produced by the structural and cultural PCA models were included in logistic regression models with the outcomes variable “likely to be well”, with adjustment for gender only. Age was excluded, as the survey included only a small age range.

## 3. Results

Characteristics of the population are presented in Table 1. The mean age of adolescents was 14 years (SD 1.7; range 11–17 years), with similar proportions of adolescents across school levels 7, 9 and 11. There were more females than males and similar numbers of participants from the major city and other locations.

Adolescents reported strong family connectedness, with most talking to or seeing extended family or feeling close to their mum or dad. In terms of community, one in three participants reported growing up in an Aboriginal community, and just over half had contact with the Aboriginal community. However, only a section of the population spoke an Aboriginal language (22%) or recognized an area as their homeland or traditional country (29%). Although participation in individual cultural activities was not high in the 12 months prior, 62.5% of adolescents had participated in at least one contemporary cultural activity—either NAIDOC, ceremonies, sporting carnivals, festivals, sorry business, or a traditional activity such as hunting, fishing, or gathering bush foods.

For structural determinants, the historical and political contexts of ongoing government intervention were revealed in the proportions reporting being Stolen Generations (17.6%); having a parent forced from their homeland (12.0%); having a parent taken by the government, a mission, or welfare (10.5%); harassment or negative contact with police (9.3%); and removal from family by community services or/and ACCA (6.8%). Social contexts of negative school environments were revealed with just over half reporting experiences of racism at school (52.0%). Not all schools gave help for adolescents to feel comfortable, identified goals with students, or set up plans with students so they could achieve their goals. The acknowledgment and inclusion of Aboriginal culture in the curriculum and school involvement in activities such as NAIDOC was low (28.4%). For the socioeconomic determinants, just under half of adolescents reported high housing transition (40.9%) and just over half having medium or low family affluence score (52.3%). Almost all youth reported geographic access to basic services, and the vast majority felt they could access a GP if needed.

### Health Outcomes

Characteristics of adolescents and bivariate associations with “likely being well” are reported in Table 2. Half (51.2%) of adolescents were coded as “likely being well”, while 14.3% were coded as likely having “mild”, 17.9% “moderate”, and 16.7% as “severe” psychological distress disorder. For demographic variables there were no statistically significant associations with “likely being well”. For cultural determinants bivariate analysis revealed statistically significant associations between “likely being well” and feeling close to dad (*p* < 0.01). Bivariate associations for structural determinants revealed statistically significant associations between “likely being well” and not having parents forced from their country (*p* = 0.03) and feeling they could access healthcare (*p* = 0.04). Associations were revealed between “likely being well” and schools giving help for adolescents to feel comfortable, adolescents not experiencing racism at school, and either parent not being taken by the government, a mission, or welfare, but these associations were not statistically significant.

The cultural PCA produced 4 components from the 11 cultural variables modelled (Table 3). These explained 64.3% of the variance in the population. These were labelled based on latent clustering of variables: (i) **cultural component 1—grew up in Aboriginal family/community and connected** (grew up in in an Aboriginal community, contact with Aboriginal community, grew up in an Aboriginal family, sees members of Aboriginal community, has talked to relatives/Elders about history, has attended Aboriginal early years program, recognizes traditional country, and has gone to Aboriginal sports carnivals); (ii) **cultural component 2—traditional cultural activities, sport, and early years** (traditional cultural activities, attended Aboriginal early years, sees members of Aboriginal community, has gone to any Aboriginal Sports carnivals); and (iii) **cultural component 3—disconnected from community, country, and culture** (has participated in NAIDOC week (inverse), has participated in Aboriginal festivals (inverse), has participated in Aboriginal sports carnivals (inverse), recognizes country (inverse), grew up in an Aboriginal community (inverse), and talks with/sees members of Aboriginal community (inverse)). Cultural component 1 explained a large proportion of variance (41.2%).

The structural PCA produced 4 components from the 11 structural variables modelled (Table 4). These explained 61.1% of the variance in the population. These were (iv) **structural component 1—institutionally imposed family displacement** (either parent taken by government/mission/welfare, either parent forced from homelands, Stolen Generations); (v) **structural component 2—Negative school environment** (school discusses goals with Aboriginal adolescent (inverse), school sets plans to achieve goals (inverse), experienced racism at school, Stolen Generations); (vi) **structural component 3—negative police contact and poverty** (family affluence scale (inverse), harassed or negative contact with police); and (vii) **structural component 4—removed from parent with positive school environment** (under care of community services/ACCA, school gives help to feel comfortable, school includes Aboriginal activities, Stolen Generations). Variance was explained equally across variables, with structural component 1 explaining most (19.4%) and structural component 4 the least (11.4%).

In looking at associations between each of the three cultural components and the four structural components, “growing up in an Aboriginal family/community and connected” associated with higher “institutionally imposed family displacement” (*p*-value = 0.011) and higher “removed from parent with positive school environment” (*p*-value = 0.012), while “disconnected from community, country, and culture” associated with lower “institutionally imposed family displacement” (*p*-value < 0.001), indicating that those disconnected still had family together.

Logistic regression modelling of PCA components revealed that for adolescents, “institutionally imposed family displacement” associated with significantly lower odds of “likely being well” (*p*-value **<** 0.05) **(**Table 5). A similar association was also revealed for “negative contact with police and poverty” and “likely being well”, but this was not statistically significant (*p* = 0.07). Conversely, adolescents who “grew up in Aboriginal family/community and connected” had statistically significant higher odds of “likely being well”.

## 4. Discussion

This collaborative co-analysis approach to social-epidemiology identified structural and cultural determinants that are “upon the people” from the insider perspective of Aboriginal people and organizations in Victoria. Modelling social survey items identified as important and unique to Aboriginal populations revealed that the relational cultural determinants of growing up in an Aboriginal family and community associated with “likely being well”, as did closeness to dad in bivariate analysis. Structural environments characterized by generational child removal and family displacement were associated negatively with “likely being well”. Bivariate analysis revealed an association with health access and “likely being well”.

In terms of structural determinants, the trauma caused by generational child removal and experiences with the justice system are well established and also acknowledged by the Australian government with the seminal “Bringing them home report” and the report of the Royal Commission into Aboriginal Deaths in Custody [44,45]. Here our data demonstrate that these structural issues continue to have ongoing impacts on the lives of school-aged adolescents as young as 11 years in Victoria in 2009, some 10–20 years after these reports were published.

The Australian Institute of Health and Welfare (2018) reported on the multiple adverse health, cultural and socioeconomic outcomes that associate with being a member of the Stolen Generations (removed before 1972) or a descendant of the Stolen Generation [46]. In Australia, official policies from 1869 to the early 1970s saw over 100,000 Aboriginal children removed from their families and placed in government and church institutions or with white families. In present times, Noongar academic Jacynta Krakouer has described ongoing high Aboriginal child removal rates as a continuum of the Stolen Generation [47]. In our study, 17.6% of adolescents connected to the identity of being Stolen Generations, and 7% reported being in the care of community services or an Aboriginal Child Care Agency. These high rates of family displacement are understood against Victorian state-wide child protection data that showed 16% of Aboriginal youth under the monitoring of child protection services, eight times the 2% of non-Aboriginal people [46]. However, despite historic and ongoing disruptions to family units, a vast majority of Aboriginal adolescents reported strong and close relationships with family, including seeing extended family and reporting closeness with either mum or dad. The maintenance of these relationships is revealing of the resilience of Aboriginal family units. The key finding that growing up in and being connected to Aboriginal family and community was associated with far greater odds (126% more likely) of “likely being well” speaks to the important role Aboriginal family has in contributing to health, especially to adolescents.

Shockingly, half of all Aboriginal adolescents in Victorian schools reported experiencing racism at school. This proportion is higher than the 34.6% of 12–17 year olds reporting racism in any setting 20 years earlier in a Victorian Aboriginal Health Service survey [48]. The interpersonal accounts of racism reported in schools, alongside a low proportion of schools including Aboriginal culture or activities in the curriculum, highlighted the problematic nature of the Victorian school system. Although our findings were inconclusive regarding the association between self-reported racism and “likely being well”, they were broadly consistent with the influence of racism in schools. Bangerang/Wiradjuri Elder and cochair of the First People Assembly of Victoria Aunty Geraldine Atkinson framed changes needed in schools as “Aboriginal staff, Aboriginal culture and Aboriginal languages taught in these schools so we can make them acceptable to all our children” [49], while research elsewhere in Australia highlighted the importance of a social justice perspective, culturally inclusive curricula, culturally differentiated quality teaching, and a primary focus on students’ wellbeing for school environments to support social and academic outcomes for Aboriginal adolescents [50]. Success in these areas is likely achieved when Victorian schools work in partnership with Aboriginal organizations that can provide leadership on culturally safe learning environments for all students. We recognize that a 2021 announcement by the Victorian government to adapt the Australian curriculum and incorporate First Nations histories, knowledges, and experiences of colonization is a step in the right direction. Further, recent work led by Distinguished Professor Marcia Langton under the Indigenous Knowledge Resources for Australian School Curricula Project is leading the way in providing resources for teachers so they are empowered to teach to Aboriginal histories.

Absolute or relative socioeconomic disadvantage in terms of employment, education, and housing are widely promoted and accepted as social determinants in Australian and global settings [51]. These determinants largely relate to everyone and have been an emphasized focus of social policy relating to Aboriginal people for decades. Here we saw 52% of Aboriginal participants as having a high family affluence score compared to 70% of participants from the wider HOWRU survey [32]. For Aboriginal populations, responding to poverty is not straightforward. It is important to understand that pathways to affluence and socioeconomic opportunities are not equal for Aboriginal populations, owing to the processes of colonization and the racialized structuring embedded within Australian society. These processes see Aboriginal people more likely to be excluded from social privilege and employment, marginalized from resources (including own lands) and opportunities, and inheriting socioeconomic deprivation [52]. Although family affluence is a measure of material poverty for both dominant and Aboriginal populations, it is important to recognize that the concept of “high affluence”, though it at some level measures the ability to meet basic human needs, it also is reflective of the aspirations of the dominant capitalist culture [53]. Particularly in the HOWRU survey, where the components of family affluence included the familial ownership of multiple cars and computers, frequent holidays, and house size, this metric speaks less to the ability to meet basic needs than social measures of adequate housing and access to foods or services would. These are imposed aspirations of Western capitalism, and there is a need for more appropriate indicators to be used in surveys relating to Aboriginal populations. We suggest future surveys consider material poverty in terms of human need rather than dominant aspirations; such a metric would have greater utility across all populations.

As we report here on the quantifiable associations between psychological distress and child removal, negative school environments, police harassment, and material poverty, we are reminded by Johnson et al. (2013) and Doyle et al. (2016) and their work with Aboriginal communities in the Goulburn–Murray Rivers region that structural determinants are real and legitimate points for health intervention [54,55]. Interventions and policy need to move beyond just responding to Aboriginal disadvantage through setting targets for individuals and communities to examining and measuring change in dominant institutions including schools, police, and social services.

For cultural determinants, expressions of Aboriginal culture are well and alive in Victoria. Here we saw that despite the continuing impacts of colonization, more than half of Aboriginal adolescents engaged in an Aboriginal cultural activity. We also saw Aboriginal adolescents as connected to friends, community Elders, and culture. Many Aboriginal adolescents in the study also participated in traditional cultural activities of hunting, fishing, or gathering bush foods, while others participated in more contemporary expressions of Aboriginal social life including sports carnivals and NAIDOC. These contemporary activities are all revealing of Aboriginal culture as dynamic and evolving. These local understandings of culture in Victoria are contemporary constructs that move beyond the limiting stereotypes of the “traditional”, the “dysfunctional”, and the “pathogenic” that have plagued epidemiology [56].

In terms of culture, our data spoke to the importance of the relational aspect of belonging to and participating in a living social system. We found that growing up in and being connected to Aboriginal family/community was associated with greater odds of “likely being well”. The high loading of Aboriginal family (0.828) for this component revealed its contribution to the association with “likely being well”. These findings highlighted the resilience of Aboriginal families and communities that have maintained identity and connection to country in a region that has a brutal colonial history. They also evidence the important work of Aboriginal organizations in Victoria, which are known to connect Aboriginal people to community and place [57]. These descriptions of culture are also consistent with those generated by Victorian Aboriginal researcher Dr Cammi Murrup-Stewart (2020), whose yarning work with young people spoke to the relational nature of culture for Aboriginal young people [58].

We found those “disconnected from community, country, and culture” had higher odds of “likely being well”, but this association was not statistically significant. We also found that “Disconnected from community, country, and culture” associated with lower “institutionally imposed family displacement” (*p*-value < 0.001), so low rates of generational child removal may reflect in “likely being well” for those “disconnected from community, country, and culture”. Although this group stated that they were not connected to Aboriginal community, they may have misinterpreted the question due to a lack of explanation. In all likelihood, many of these participants would be connected to an Aboriginal family (we know that most Aboriginal children are raised by an Aboriginal parent or kin), and this may be why they are “likely to be well”. However, we are also mindful that the small sample size makes it difficult to determine if this is true beyond the study. The interpretation of Aboriginal organizations was integral to understanding why “disconnected from community, country, and culture” associated (although not significantly) with “likely being well”. The coauthors from Aboriginal organizations spoke of the process of colonization in Victoria and how it worked to disperse, dislocate, and make less visible Aboriginal people. Individuals who are disconnected from community are less visible, and with that can come greater access to opportunities (school, employment) and less exposure to racism. For example, participation in community activities such as marches (i.e., NAIDOC) or in Aboriginal sporting teams, although acknowledged as important from the relational aspect of connecting people and building Aboriginal identity, made individuals more visible as Aboriginal and exposed to interpersonal racism [59].

For cultural determinants, speaking language and knowing homeland were not widely reported by adolescents, reflective of the aggressive nature of colonization in Victoria a history of forced assimilation and mass displacement. However, this will be an important indicator to monitor given the proliferation of Aboriginal language revitalization programs in Victoria and the development of other cultural heritage programs and activities since this survey was administered.

Our analysis was only possible because the HOWRU Survey was designed to include an Aboriginal module. We emphasize the important need for social surveys to have measures that capture the social realities of Aboriginal people as identified by them. We also propose that an ecosocial theory and a multilevel framework that focuses on “who” and “what” drives social inequities, rather than focusing on individuals or communities, is a useful approach for social epidemiology involving Aboriginal populations [60]. Work by the Victorian Aboriginal Health Service two decades ago spoke to the importance of ecological frameworks as alternatives to the linear frameworks traditionally used in non-communicable-disease epidemiology. Thompson et al. (2000) called for ecological approaches that recognized structural issues when they wrote, “The advantage of applying this [ecological] approach to epidemiological models is that it enables a more sophisticated and comprehensive understanding of risk and allows for the identification of factors that reflect people’s own constructions of their social worlds through naturalistic observation” [21] (p. 1461).

A maintained focus on dominant social determinants in social-epidemiology has come from a scientific methodology that has centred and normalized Western epistemologies (knowledges) and axiologies (ideologies) [61]. By not engaging with Aboriginal-defined social determinants, the discipline of epidemiology applies a casualness to the construction of Aboriginal social worlds that is at odds with a scientific discipline that focuses on statistic precision and rigor [62]. Further, by overlooking cofounding—the basic problem of compatibility between Aboriginal and non-Aboriginal groups—questions are raised about the internally and externally validity of these analyses. As highlighted by Larkins (2006), research relating to Aboriginal people, in general, is methodologically strengthened through the inclusion of Aboriginal people and their knowledges and the centring of these voices [61]. Rigney describes a fundament of Indigenist research is that it privileges Aboriginal voice [63]. Here, Aboriginal voice is reflected not only in the authorship by Aboriginal academics, but also in participation by Aboriginal organizations that speak firsthand about their social world and that of the people they work in proximity with. Another fundament of Indigenist research is resistance—of developing alternative discourses to those created without Aboriginal people [63]. We deliberately sought to resist the normalization of dominant sociocultural experiences and lives and to highlight that sociocultural determinants are different, as are health causality pathways. We know and reveal here that health has never been a consequence of Aboriginal people’s inability to meet parity for a series of dominant social concepts [64].

### Strengths and Limitations

A key strength of this paper is its process, which deliberately sought to centre Aboriginal ways of knowing, doing, and being within social-epidemiology. Co-analysis meant that rather than relying on assumptions in the literature around social determinants, the community the data related to was able to articulate the social contexts and social processes that happen in its social world. This acknowledged the constructionist nature of epidemiological knowledge, but also the expert voices of Aboriginal people and organizations. As highlighted by Brough et al., epidemiology is not a value-free science and embedded within are the understandings, values, and positioning of those who create it [1]. Aboriginal identities and sociocultural conditions can be only partially known through dominant frameworks that provide one worldview and interpretation of the world. Aboriginal people too have legitimate understandings and interpretations of their identities and sociocultural realities [65].

Here, Aboriginal voices have asked fundamentally different social questions of the data than researchers of dominant Victorian institutions have asked in the past [66,67]. Rather than asking how we are different to non-Aboriginal people, by examining social determinants of dominant populations, it was asked, what are the unique social determinants specific to Aboriginal health? This frame was only possible because the dataset was available. Regrettably, when the Victorian Department of Education and Training conducted subsequent health and wellbeing surveys in Victorian schools in 2014 and 2018, they did not include an Aboriginal module. This means that health and social dimensions unique to the live of Aboriginal adolescents were not captured. We make the strong recommendation that future health and social surveys that collect an Aboriginal identifier must also collect health and social data that most accurately describes the social worlds of Aboriginal participants. These measures are best developed by or with Aboriginal organizations [28].

In recognizing key strengths of this research, we are also mindful of its limitations—in particular, the poor statistical power that came from a small sample size. In general, PCA components for small datasets are not very generalizable to the wider population, but the high loadings on components (>0.800) we saw here suggests that components were likely revealing of the latent structure in the wider community [42]. This limited sample size was a consequence of the sampling method, which selected for equal proportions of youth by area and school type and overlooked how this would impact Aboriginal participation. We recommend all future surveys aim to achieve greater power through targeted sampling of schools with Aboriginal students or conversely through Aboriginal networks or organizations. With larger sample size, a more nuanced exploration of health and its structural and cultural determinants for subpopulations can be conducted.

Time has also elapsed since the study; however, we believe the findings are still relevant, as many of the structural issues, particularly those relating to child removal and policing, have in fact intensified.

In interpreting findings, we realize that the HOWRU Aboriginal population is unlikely to be representative of all Aboriginal adolescents in Victoria. Firstly, there are adolescents who may not be comfortable reporting Aboriginal status in a government survey. Secondly, the survey relates to adolescents attending school, so it does not capture all Aboriginal adolescents. Data for 2010, the year after this survey was conducted, reveals that retention rates for students in years 10 to 12 was 50.9% for Aboriginal students, and it is likely that those not in school have different health and social profiles [68]. For example, it is likely that prevalence estimates of experiences of racism in school are likely to be underestimated, as racism associates with school disengagement and nonattendance [69].

Quantitative survey data is also unlikely to capture Aboriginal culture or structures and mechanisms of Australian society in their full complexity. In assigning sociocultural phenomena to a series of questions and numbers, we may not be measuring what we think we are [70]. We also realize there is a tendency of quantitative research to essentialize culture. We know that the Victorian population is heterogenous and that there are many expressions of Aboriginal culture and many encounters with the dominant culture, for these adolescents are all cultural beings. We know that the HOWRU Survey data did not include measures for Aboriginal cultural constructs of family, identity, and self-determination, which were identified as cultural determinants in the Victorian literature and discussed in early meetings with the collaborative. Future design of an Aboriginal module should consider indicators that capture these cultural concepts. We were also limited by the measure we used to capture “Aboriginal health and wellbeing”. Although the K-10 has been used by others and has excellent agreement with a culturally modified K-5, it is a universal rather than Aboriginal-specific measure of health [71]. It is also a measure of distress rather than wellbeing. Since 2009, much work has been done to develop Aboriginal measures of health, and there are now measures such as the Aboriginal Risk and Resilience Questionnaire developed by Dr Graham Gee and the Victorian Aboriginal Health Service that could be used in present-day surveys to measure health and wellbeing [72].

We could not ascertain from the data if observed relationships were causal because of the cross-sectional nature of the HOWRU Survey. However, there is already strong evidence by way of art, music, protests, biography, and qualitative accounts that colonization characterized by generational displacement, child removal, policing, and racism is bad for Aboriginal health [12]. All our study did was quantify this association in a palatable, empirical form that Western policymakers will see as “evidence”. We are also mindful that expressions of health and cultural or structural determinants can change across the life course, and associations reported here would need to be tested via longitudinal study to empirically demonstrate that structural and cultural determinants impact on health. We also cannot determine the intensity of social–cultural phenomena. Our conceptual framework is also one-dimensional and excludes time and space on health, aside from the inclusion of history as a contextual structural determinant.

## 5. Conclusions

Here Aboriginal voices contributed to epidemiology through a co-analysis process that determined “who” and “what” is upon Aboriginal people. Using a co-analysis approach, the voices of Aboriginal researchers and Aboriginal organizations were able to construct a social world that aligned more closely with their ways of knowing, doing, and being. The resulting co-analyses revealed that growing up in an Aboriginal family is likely the biggest cultural support for Aboriginal adolescents “likely being well”, while past and present structural issues of ongoing institutionalized family dislocation and removal, negative contact with police, material poverty, exclusionary school programs and curriculums, and high rates of racism in schools are likely “what is upon” Aboriginal adolescents aged 14–17 years. These structural factors are all legitimate points for health intervention to improve health outcomes for Aboriginal adolescents, but they require Victorian systems to change rather than Aboriginal people or communities.

## Figures and Tables

**Table 1:** Frequency of demographic, structural, and cultural characteristic identified by the collaborative (*n* = 88).

Variable	*n* *	%	(95% CI)
Demographic			
Year level			
Year 7	28	31.8	(22.1–41.5)
Year 9	34	38.6	(28.5–48.8)
Year 11	26	29.5	(20.0–39.1)
Male	38	43.2	(32.8–53.5)
Major-city	47	53.4	(43.6–64.5)
Cultural determinants			
Talked to Elders or relatives about history or culture	52	65.8	(55.4–76.3)
Talk with/see members of community	55	68.8	(58.6–78.9)
Talk with/see members of extended family	80	95.2	(90.7–99.8)
Speak Aboriginal language	17	21.5	(12.5–30.6)
Aboriginal values	54	68.4	(58.1–78.6)
Recognize country	25	29.4	(19.7–39.1)
Grew up in an Aboriginal family	38	44.7	(34.1–55.3)
Grew up in an Aboriginal community	25	29.4	(19.5–38.7)
Contact with Aboriginal community	46	54.1	(43.5–64.7)
Uses Aboriginal organizations	16	18.2	(10.1–26.2)
Participates in Aboriginal ceremonies	12	13.6	(6.5–20.8)
Participates in Aboriginal Sports carnivals	21	23.9	(15.0–32.8)
Participates in Aboriginal festivals	16	18.2	(10.1–26.2)
Participates in NAIDOC week	15	17.0	(9.2–24.9)
Participates in Sorry business	10	11.4	(4.7–18.0)
Participates in traditional cultural activity	35	40.2	(29.9–50.5)
Attended Aboriginal Early years program	20	28.2	(17.7–38.6)
Aboriginal friends	39	52.0	(40.7–63.3)
Feels close to mum	71	82.6	(74.5–90.6)
Feels close to dad	57	75.0	(65.3–84.7)
Structural determinants			
School gives help to feel comfortable	47	64.4	(53.4–75.4)
School identifies goals with Aboriginal adolescent	34	47.2	(35.7–58.8)
School sets plans to achieve goals	25	34.7	(23.7–45.7)
School includes Aboriginal activities and culture in curriculum	21	28.4	(18.1–38.7)
Experienced racism at school	39	52.0	(40.7–63.3)
High housing transition (>3 times)	36	40.9	(30.6–51.2)
Police harassment or negative contact	7	9.3	(2.7–15.9)
Low or medium family affluence	46	52.3	(41.8–62.7)
Stolen Generations	15	17.6	(9.5–25.8)
Either parent taken by gov/mission/welfare	9	10.5	(4.0–16.9)
Either parent forced from homelands	10	12.0	(5.0–19.1)
Feels they can access general practitioner	72	94.7	(89.7–99.8)
Under care of community services/ACCA	6	6.8	(1.6–12.1)
Access to basic services	72	92.3	(86.4–98.2)

* Not all demographic, structural, and cultural variables were complete for all 88 participants.

**Table 2:** Bivariate analysis of demographic and social characteristics and likely being well (Kessler-10 score 10–19).

	*n*	%	95%CI	RR	95%CI	*p*-Value
Demographic						
Year 7	14	56.0	36.5–75.5	1.21	0.62–2.36	0.43
Year 9	14	42.4	25.6–59.3	0.70	0.41–1.21	
Year 11	15	57.7	38.7–76.7	1.30	0.68–2.49	
Male	20	54.1	38.0–70.1	1.12	0.69–1.82	0.64
Female	23	48.9	34.6–63.2	0.91	0.63–1.33	
Major-city	22	47.8	33.4–62.3	0.89	0.61–1.32	0.57
Regional	20	54.1	38.0–70.1	1.15	0.71–1.86	
Cultural determinants						
Talked to Elders or relatives about history or culture	26	50.0	36.4–63.6	0.95	0.69–1.30	0.75
Talks with/see members of community	26	48.1	34.8–61.5	0.95	0.71–1.29	0.75
Talks with/see members of extended family	40	51.3	40.2–62.4	1.05	0.95–1.16	0.31
Speaks Aboriginal language	8	47.1	23.3–70.8	0.80	0.35–1.90	0.65
Aboriginal values	30	56.6	43.3–69.9	1.21	0.89–1.64	0.22
Recognizes country	11	45.8	25.9–65.8	0.87	0.53–1.44	0.58
Grew up in an Aboriginal family	19	52.8	36.5–69.1	1.09	0.67–1.79	0.73
Grew up in an Aboriginal community	14	58.3	38.6–78.1	1.37	0.69–2.72	0.37
Has contact with Aboriginal community	23	51.1	36.5–65.7	1.05	0.71–1.55	0.82
Uses Aboriginal organizations	10	62.5	38.8–86.2	1.59	0.64–3.98	0.31
Participates in Aboriginal ceremonies	6	54.5	25.1–84.0	1.14	0.38–3.46	0.81
Participates in Aboriginal Sports carnivals	10	47.6	26.3–69.0	0.87	0.41–1.82	0.71
Participates in Aboriginal festivals	8	50.0	25.5–74.5	0.95	0.39– 2.30	0.92
Participates in NAIDOC week	7	46.7	21.4–71.9	0.83	0.33–2.09	0.70
Participates in Sorry business	4	40.0	9.6–70.4	0.64	0.19–2.09	0.45
Participates in traditional cultural activities	19	55.9	39.2–72.6	1.24	0.73–2.09	0.42
Attended Aboriginal Early years program	13	65.0	44.1–85.9	1.56	0.71–3.44	0.26
Aboriginal friends	23	62.2	46.5–77.8	1.43	0.89–2.31	0.13
Feels close to mum	38	55.9	44.1–67.7	1.18	0.96–1.45	0.11
Feels close to dad	32	60.4	47.2–73.5	**1.52**	**1.13–2.06**	<0.01 *
Structural determinants						
School gives help to feel comfortable	29	61.7	47.8–75.6	1.36	0.95–1.96	0.08
School discusses goals with Aboriginal adolescent	19	55.9	39.2–72.6	1.04	0.64–1.70	0.88
School sets plans to achieve goals	12	48.0	28.4–67.6	1.27	0.59–2.73	0.39
School includes Aboriginal activities and culture in curriculum	13	61.9	41.1–82.7	1.34	0.63–2.84	0.44
Experienced racism at school	17	43.6	28.0–59.2	0.66	0.42–1.02	0.06
High housing transition	19	52.8	36.5–69.7	1.06	0.69–1.60	0.80
Harassed or negative contact with police	2	28.6	0.0–62.0	0.34	0.07–1.64	0.16
Low or medium family affluence	25	59.5	44.7–74.4	1.40	0.90–2.18	0.13
Stolen Generations	8	53.3	28.1–78.6	1.06	0.42–2.66	0.90
Either parent taken by gov/mission/welfare	2	22.2	0.0–49.4	0.28	0.06–1.26	0.07
Either parent forced from homelands	2	20.0	0.0–44.8	0.28	0.05–1.03	0.03 *
Feel they can access general practitioner	37	53.6	41.9–65.4	**1.13**	**1.00–1.26**	0.04 *
Under care of community services/ACCA	3	60.0	17.1–100	1.43	0.25–8.13	0.68
Access to basic services	38	54.3	42.6–66.0	1.07	0.93–1.22	0.32
Total	43	51.2	40.5–61.9			

Bold text denotes magnitude of association is significant * denotes that characteristic differs at the *p* < 0.05 level for those “likely being well” and those with “likely to have psychological distress”.

**Table 3:** Principal component analysis of cultural determinants (*n* = 68; includes all participants with a complete profile).

	(i) Cultural Component 1: Grew Up in Aboriginal Family/Community and Connected	(ii) Cultural Component 2: Traditional Cultural Activities, Sports, and Early Years	(iii) Cultural Component 3: Disconnected from Community, Country, and Culture
Grew up in an Aboriginal community	**0.847**	−0.114	**−0.439**
Contact with Aboriginal community	**0.834**	0.036	−0.374
Grew up in an Aboriginal family	**0.828**	0.030	−0.385
Talks with/sees members of community	**0.711**	**0.450**	**−0.598**
Talked to Elders or relatives about history or culture	**0.564**	0.275	−0.357
Attended Aboriginal Early years program	**0.561**	**0.550**	−0.200
Participates in Traditional cultural activity	−0.094	**0.778**	0.080
Participates in NAIDOC week	0.387	0.115	**−0.870**
Participates in Aboriginal festivals	0.314	−0.073	**−0.805**
Participates in Aboriginal sports carnivals	**0.407**	**0.599**	**−0.670**
Recognizes country	**0.473**	0.030	**−0.629**

Bold text denotes a loading > 0.400 indicating a cultural determinant, groups with a component.

**Table 4:** Principal component analysis of structural determinants (*n* = 68; includes all participants with a complete profile).

	(iv) Structural Component 1: Institutionally Imposed Family Displacement	(v) Structural Component 2: Negative School Environment	(vi) Structural Component 3: Negative Police contact and Poverty	(vii) Structural Component 4: Removed from Parent with Positive School Environment
Either parent taken by gov/mission/welfare	**0.907**	−0.010	−0.006	0.094
Either parent forced from homelands	**0.888**	−0.044	−0.074	−0.017
Stolen Generations	**0.483**	**0.433**	0.278	**0.429**
School discusses goals with Aboriginal adolescent	0.092	**−0.801**	0.055	0.170
School sets plans to achieve goals	0.178	**−0.764**	0.139	0.233
Experienced racism at school	0.332	**0.432**	−0.165	0.188
Harassed or negative contact with police	0.035	−0.048	**0.738**	0.007
Low or medium family affluence scale	0.158	0.208	**−0.683**	−0.148
Under care of community services/ACCA	0.106	0.021	0.139	**0.756**
School gives help to feel comfortable	−0.056	−0.275	−0.386	**0.626**
School includes Aboriginal activities and culture in curriculum	−0.167	−0.384	0.287	**0.460**

Bold text denotes a loading > 0.400 indicating a structural determinant, groups with a component.

**Table 5:** Odds ratio of “likely being well” associated with components from PCA for Aboriginal adolescents from Victoria.

Component	Odds Ratio	95%CI	*p*-Value
Cultural component 1—grew up in Aboriginal family/community and connected	2.26	**(1.01–5.06)**	0.046 *
Cultural component 2—traditional cultural activities, sport and early years	0.91	(0.48–1.73)	0.773
Cultural component 3—disconnected from community, country and culture	1.75	(0.85–3.58)	0.127
Structural component 1—institutionally imposed family displacement	0.49	**(0.24–0.97)**	0.040 *
Structural component 2—negative school environment	1.05	(0.54–2.04)	0.885
Structural component 3—negative police contact and poverty	0.53	(0.26–1.06)	0.073
Structural component 4—removed from parent with positive school environment	1.51	(0.78–2.89)	0.220
Gender	0.99	(0.28–3.50)	0.988

Bold text denotes that magnitude of association is significant; * denotes that characteristic differs at the *p* < 0.05 level for those “likely being well”.

## Data Availability

Data were obtained from the Victorian government Department of Education and Early Childhood Development, which is custodian of the HOWRU survey data.

## Appendix A

**Table A1:** Collection and measurement of cultural determinants.

Variable	Question/s	Responses	Dichotomised Variable
Aboriginal module			
Talked to Elders/relatives about history or culture	In the past year, how often did you talk with older relatives or Elders about Aboriginal or Torres Strait Islander history or culture?	Rarely/sometimes/often/very often/never	Yes (rarely, sometimes, often, or very often)/no (never)
Talk with/see members of community	See other members of your Aboriginal or Torres Strait Islander community?	Almost every day/2–3 times a week/once a week, once a month, rarely/never	Yes (Almost every day, 2–3 times a week, once a week, or once a month)/no (never)
Talk with/see members of extended family	See or talk with members of your extended family? (e.g., cousins/aunts/uncles/nieces/nephews)	Almost every day/2–3 times a week/once a week, once a month, rarely/never	Yes (Almost every day, 2–3 times a week, once a week, or once a month) /no (never)
Speak Aboriginal language	Do you speak any other Aboriginal or Torres Strait Islander language?	Yes/Yes, some words/No	Yes/no (Yes, some words coded as yes)
Aboriginal values	Are Aboriginal or Torres Strait Islander values important to you, such as respect for your elders, cultural traditions, and connection to country and lore?	Somewhat/very important/not at all important	Yes (somewhat or very important)/no (not at all)
Recognize country	Do you recognize an area as your homelands or traditional country?	Yes/no (no/don’t know)	
Grew up in an Aboriginal family	Did you grow up in an Aboriginal or Torres Strait Islander family?	Yes/no	
Grew up in an Aboriginal community	Did you grow up in an Aboriginal or Torres Strait Islander Community?	Yes/no	
Contact with Aboriginal community	Do you have contact with an Aboriginal or Torres Strait Islander Community?	Yes/no	
Uses Aboriginal organizations	In the last 12 months, have you gone to any Aboriginal or Torres Strait Islander: Or been involved with any Aboriginal or Torres Strait Islander organizations?	Yes/no	
Participates in Aboriginal ceremonies	In the last 12 months, have you gone to any Aboriginal or Torres Strait Islander: Ceremonies?	Yes/no	
Participates in Aboriginal Sports carnivals	In the last 12 months, have you gone to any Aboriginal or Torres Strait Islander: Sports carnivals?	Yes/no	
Participates in Aboriginal festivals	In the last 12 months, have you gone to any Aboriginal or Torres Strait Islander: Festivals or carnivals involving arts, craft, music, or dance?	Yes/no	
Participates in NAIDOC week	In the last 12 months, have you gone to any Aboriginal or Torres Strait Islander: NAIDOC week activities?	Yes/no	
Participates in Aboriginal cultural activities—sorry business	In the last 12 months, have you gone to any Aboriginal or Torres Strait Islander: Funerals or sorry business?	Yes/no	
Participates in Traditional cultural activity	Have you ever done any of the following? (any of hunted, fished, gathered berries)	Yes/no	
Attended Aboriginal Early years program	Did you attend an Aboriginal or Torres Strait Islander childcare centre, preschool, or kindergarten?	Yes/no (no or don’t know)	
Aboriginal friends	Which is most true of your friends?	All of my friends are Aboriginal/most of my friends are Aboriginal/some of my friends are Aboriginal/none of my friends are Aboriginal	Yes (all, some, or most)/no (none)
Wider HOWRU module			
Feels close to mum	Do you feel very close to your mother?	YES!/yes/no/NO!	Yes (YES! or yes)/no (no or NO!)
Feels close to dad	Do you feel very close to your father?	YES!/yes/no/NO!	Yes (YES! or yes)/no (no or NO!)

**Table A2:** Collection and measurement of structural determinants.

Variable	Question/s	Responses	Dichotomised Variable
Aboriginal module			
School gives help to feel comfortable	Does your school give you enough help for you to feel comfortable at school and learn what is taught?	Yes/no (no or don’t know)	
School identifies goals with Aboriginal adolescent	Have you discussed your future goals and aspirations with a teacher?	Yes/no (no or don’t know)	
School sets plans to achieve goals	Have you set up a plan to achieve your future goals and aspirations, with the help of your teacher?	Yes/no (no or don’t know)	
School includes Aboriginal activities and culture in curriculum	Does your school acknowledge and include Aboriginal or Torres Strait Islander culture in its curriculum and activities, for example, NAIDOC week activities or other activities or lessons throughout the year?	Yes/no	
Experienced racism at school	Have you experienced racism at school?	Yes/no	
Police harassment or negative contact	In the past year, have you been harassed by or had any negative contact with the police, for example, physical or verbal abuse, etc.?	Yes/no (no or not sure)	
Stolen Generation	Do you identify as belonging to the stolen generation?	Yes/no (no or don’t know)	
Either parent taken by gov /mission/welfare	Were either of your parents taken away from their family by a mission, the government or welfare?	Yes/no	
Either parent forced from homelands	Were either of your parents forced to move from an area which was your traditional country or homeland?	Yes/no	
Under care of community services/ACCA	Have you ever been under the care of community services/Aboriginal Community Control Association (ACCA)?	Yes/no (no or don’t know)	
Wider HOWRU survey			
High housing transition (higher than sample average)	How many times have you changed homes since kindergarten?	Never/1or 2/3 or 4/5 or 6/7 or more times	No (never or 1 or 2 times)/yes (3+ times)
Family affluence scale (HBSC)	4 items: Does your family own a car, van or truck? (no/yes/two);Do you have your own bedroom for yourself? (yes/no); During the past 12 months, how many times did you travel away on holiday with your family? (none, one, 2+); How many computers does your family own? (none/one/two/more than 2)	9 item scale using 4 questions	Boyce et al. (2006) where low–med (0, 1, 2, 3, 4, 5) or high (6, 7, 8, 9) [73]
Feel they can access general practitioner	Does adolescent feel that they can access GP services if needed?	Yes/no	
Access to basic services	There is access to basic services such as banks and medical clinics in my neighbourhood.	Strongly agree/agree/disagree/strongly disagree	Yes (strongly agree or agree)/no (disagree or strongly disagree)
